# Dynein light chains 1 and 2 are auxiliary proteins of pH-sensitive Kir4.1 channels

**DOI:** 10.1016/j.jbc.2025.108393

**Published:** 2025-03-10

**Authors:** Sun-Joo Lee, Jian Gao, Ellen Thompson, Jonathan Mount, Colin G. Nichols

**Affiliations:** 1 Department of Cell Biology and Physiology and the Center for Investigation of Membrane Excitability Diseases, Washington University School of Medicine, St Louis, Missouri, USA; 2 Department of Anesthesiology, Weill Cornell Medical College, New York, New York, USA

**Keywords:** Kir4.1, pH sensitivity, dynein light chain 1, LC8, auxiliary protein

## Abstract

Inward rectifier Kir4.1 potassium channels are abundantly expressed in cells that are important for electrolyte homeostasis. Dysregulation of Kir4.1 underlies various neurological disorders. Here, through biochemical and structural studies of full-length Kir4.1, we show that dynein light chain 1 and 2 proteins, also as known as LC8, copurify with Kir4.1 at stoichiometric levels. Direct interaction between Kir4.1 and LC8 is supported by *in vitro* binding assays and reiterated with native Kir4.1 proteins from mouse brain. Notably, we identify a LC8 binding motif in the unstructured N terminus of Kir4.1. Among Kir subtypes, the motif is unique to Kir4.1 and is highly conserved between Kir4.1 orthologs. Deletion of the predicted anchoring sequence (ΔTQT) resulted in loss of LC8 interaction with Kir4.1 N-terminal peptides as well as with full-length Kir4.1, suggesting that the binding site is necessary and sufficient for the interaction. Purified Kir4.1-ΔTQT mutant proteins exhibited normal channel activity *in vitro*, whereas WT proteins lost phosphoinositide-(4,5)-phosphate activation. Single-particle cryo-EM analysis of the full-length proteins revealed extremely heterogeneous particles, indicating deformation from the typical fourfold symmetric conformation. Additional electron density attached to the Kir4.1 tetramer, ascribed to an LC8 dimer, further supports the direct interaction between the two proteins. While the biological implications of this interaction await further elucidation, the strong conservation of the LC8 binding motif suggests its potential importance in the regulation of Kir4.1 channels.

Inwardly rectifying potassium (Kir) 4.1 channels, prominently regulated by phosphoinositide-(4,5)-phosphate (PIP_2_) and intracellular pH (1), are highly expressed in glial cells in the central nervous system (2, 3, 4) and kidney epithelial cells (5). They play critical roles in regulation of local and whole-body potassium levels, and hence, electrolyte homeostasis (1), and Kir4.1 loss-of-function mutations cause Seizures, Sensorineural deafness, Ataxia, intellectual (Mental) disability, and Electrolyte imbalance (SeSAME) syndrome (6), also known as EAST syndrome (7).

Interestingly, both Kir4.1 channel activity and channel surface expression are directly affected by phosphorylation of N-terminal Tyr9 (8), which is located far beyond the structured part of the protein. K_ATP_ channel cryo-EM structures determined with full-length Kir6.2 and SUR2 proteins reveal strikingly unique interactions of the “unstructured” N-terminal and intervening loops with the rest of the complex that are critical in K_ATP_ gating (9, 10). Determined with both N and C termini truncated, a recent Kir4.1 cryo-EM structure (Protein Data Bank code: 8I5M) (11) was structurally similar to the previously determined Kir6.2 PIP_2_-bound structure (12) and provides no information regarding flexible parts. In the present study, we purified full-length Kir4.1 proteins and carried out biochemical, functional, and structural characterization. Full-length Kir4.1 proteins show structurally unique features and reveal a novel and direct interaction of dynein light chain 1 (and 2), so called LC8, to the unstructured N terminus of Kir4.1. LC8 interaction had significant effects on channel activity of Kir4.1 proteins, indicating LC8 is a *bona fide* Kir4.1 auxiliary protein.

## Results

**Figure 1:**
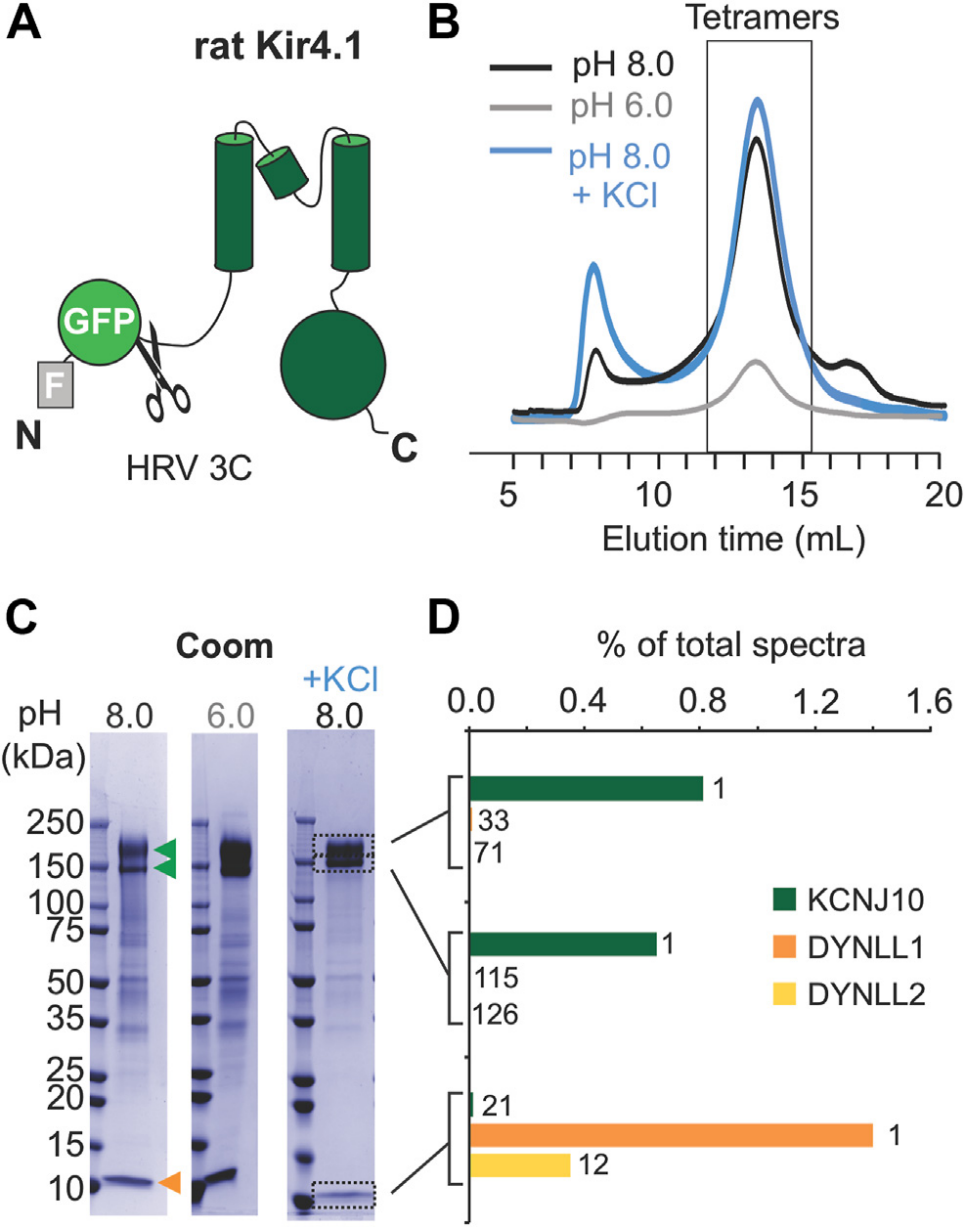
**Full-length Kir4.1 channel overexpression and purification.***A*, schematic of the Kir4.1 construct, with N-terminal FLAG and GFP tags and HRV 3C cleavage sequence. *B*, size-exclusion chromatography (SEC) profiles of purified Kir4.1 without tags, in indicated buffer conditions. *Box* indicates tetrameric fractions. *C*, 1D SDS-PAGE gel images from SEC runs in (*B*). Tetrameric Kir4.1 bands (*green arrows*) and 10 kDa copurified proteins (*orange arrows*) are indicated. Samples extracted for peptide mass spectrometry (MS) are indicated by *dotted boxes*. *D*, bar graph of percentage of KCNJ10, DYNLL1, and DYNLL2 peptides in each MS sample. Number indicates rank order of the protein in each sample. The complete lists of the spectrum analysis are in SI SF1 and ST1. DYNLL1, dynein light chain 1; DYNLL2, dynein light chain 2; HRV, human rhinovirus; Kir, inwardly rectifying potassium.

N-terminal FLAG-GFP-tagged full-length Kir4.1 was stably expressed in human embryonic kidney 293 cells (Fig. 1*A*). Tetramer peaks of purified Kir4.1 proteins overlapped for pH 8.0 and pH 6.0 buffer conditions, indicating that the complexes were stable in detergent micelles over the physiologically relevant pH range (1) (Fig. 1*B*) in which the channel goes from physiologically active (pH 8.0) to inhibited state (pH 6.0). Even though Kir4.1 protein (at molecular weights near 150 kDa after the N-terminal tags were cleaved off) was predominant, some additional bands were present in the purified proteins, particularly proteins of ∼10 kDa. These ∼10 kDa proteins were as abundant as Kir4.1 (Fig. 1*C*), at both pH 8.0 and pH 6.0, and their abundance was not substantially reduced even at high ionic strength (500 mM KCl) (Fig. 1*C*). To identify these copurified proteins, purified Kir4.1 solution samples were subject to peptide mass fingerprinting, which identified >2000 different proteins (Supporting Information (SI) SF1 and ST1). After Kir4.1 itself, the second most highly populated protein was dynein light chain 1 (DYNLL1) (SI SF 1,2 and Fig. 1*D*), and the paralog dynein light chain 2, which can form a heterodimer with DYNLL1 (SI SF 1,2 and Fig. 1*D*). The high abundance of these two small proteins (collectively referred to as LC8 proteins) matched the 1D gel image of the protein sample (Fig. 1*C*), in which bands at ∼10 kDa were most abundant next to the top two bands of Kir4.1 proteins.

**Figure 2:**
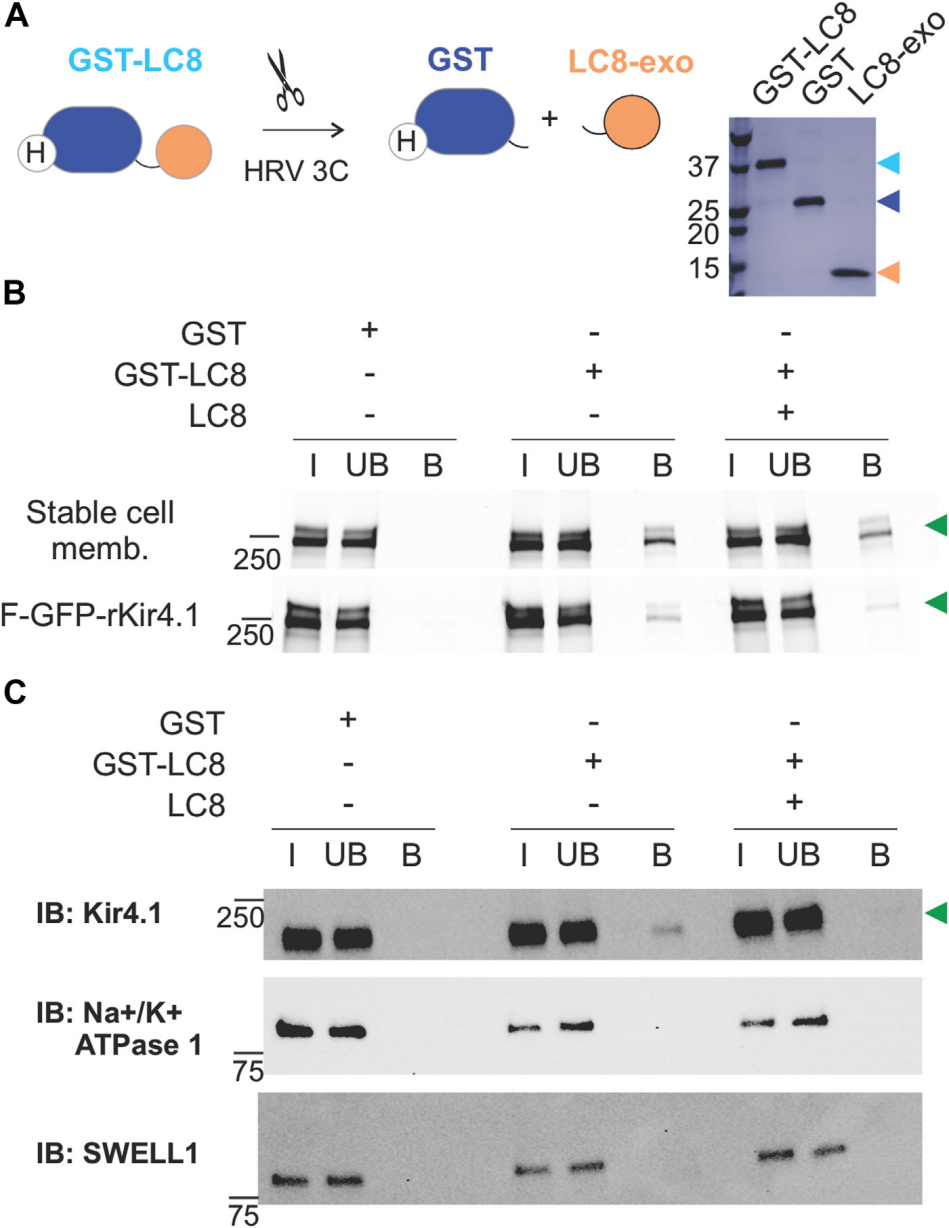
**Pull-down of Kir4.1 by GST-tagged LC8.***A*, schematics of proteins utilized for pulldown assays. Purified GST-LC8 fusion proteins were separated into free GST and LC8 by HRV 3C protease and individually purified (see the Experimental procedures section). F-GFP-Kir4.1 was used with the N-terminal FLAG and GFP tags present. *B*, pulldown of Kir4.1 from detergent solubilized membrane fraction by GST (negative control), GST-LC8, or GST-LC8 plus free LC8 was confirmed by the GFP signals. The same experiment was repeated against purified F-GFP-Kir4.1. *C*, the same experiment was repeated with detergent-solubilized mouse brain membrane fractions. Immunoblotting with anti-Kir4.1 antibodies was used to detect Kir4.1. Immunoblotting with Na^+^/K^+^ ATPase I or SWELL1 was performed. B, bound fraction; I, input; UB, unbound fraction. *Arrowheads* indicate different proteins; *Green*: F-GFP-Kir4.1; *Cyan*: GST-LC8; *Blue*: GST; *Pale orange*: exogeneous free LC8; and *Orange*: endogenous LC8. The whole gel images are shown in SI SF 3,4. GST, glutathione-*S*-transferase; HRV, human rhinovirus; Kir, inwardly rectifying potassium; LC8, light chain 8.

The small size but high abundance suggested LC8 proteins might directly interact with Kir4.1. To test this directly, pull down of Kir4.1 was performed (13) (Fig. 2*A*). First, glutathione-*S*-transferase (GST)-LC8, but not free GST, pulled down F-GFP-Kir4.1 from detergent-solubilized membrane fractions, and this effect was attenuated in the presence of added free LC8 (Fig. 2*B*). To exclude the possibility that the interaction was indirect, *via* mediating proteins, the same experiment was repeated with purified F-GFP-Kir4.1 (Fig. 2*B*), with essentially the same results, indicating that the two proteins directly interact. Finally, to test whether LC8 could interact with native Kir4.1 proteins, detergent-solubilized mouse brain membrane fractions were incubated with GST-LC8 or free GST, and native Kir4.1 was detected by immunoblotting with anti-Kir4.1 antibodies, for which specificity was confirmed (SI SF 4A) (Fig. 2*C*). Again, essentially the same results were obtained as with recombinant F-GFP-Kir4.1 proteins. Other abundant membrane proteins, including Na^+^/K^+^ ATPase I and SWELL1, were not pulled down by GST-LC8 (Fig. 2*C*). Together, these results strongly support a direct interaction between LC8 and Kir4.1 that is physiologically relevant.

**Figure 3:**
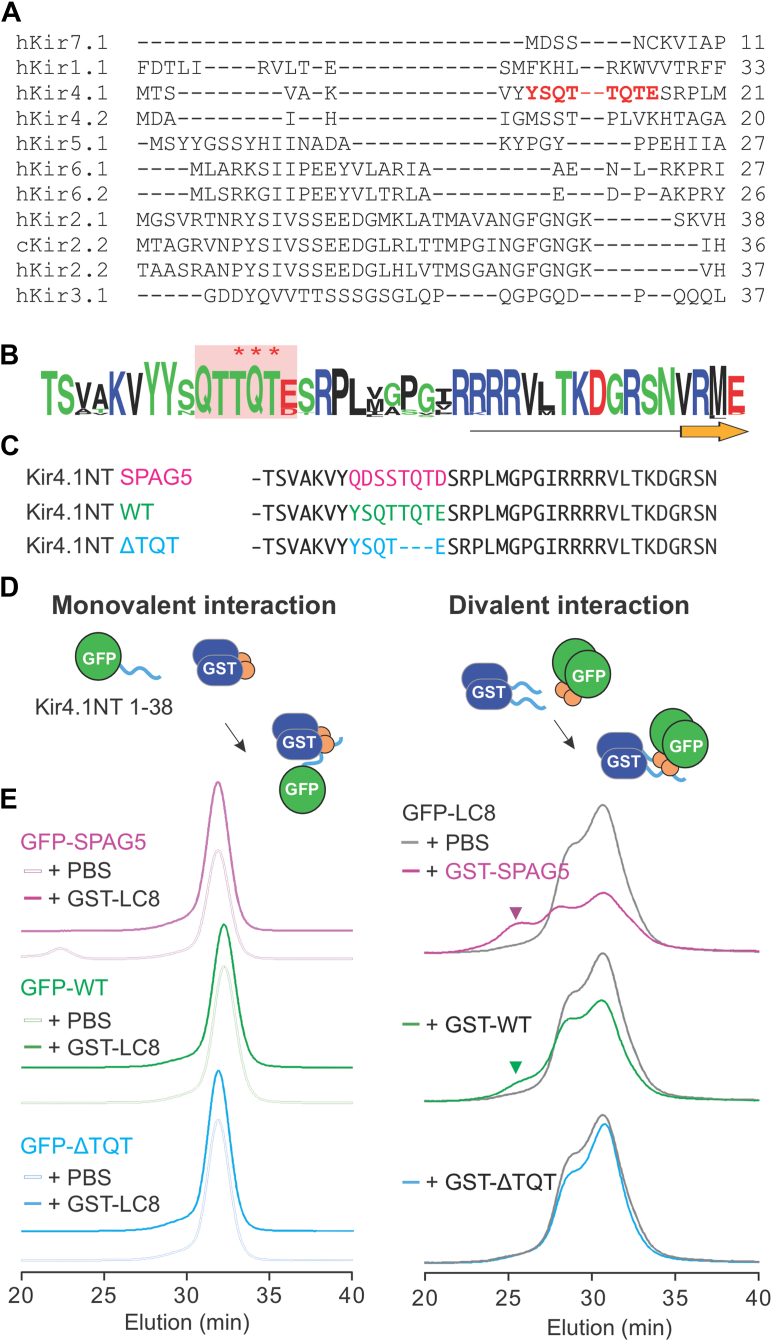
**LC8 binding motif in the N-terminal loop of Kir4.1.***A*, multiple sequence alignment of human Kir subtypes (PredLC8-predicted LC8 binding motif highlighted in *red*). *B*, amino acid sequence conservation of Kir4.1 N-terminal (40) (predicted LC8 binding motif boxed in *pink*, core TQT anchoring sequence marked by ∗∗∗). The *ribbon diagram* indicates the beginning of the structured portion of Kir proteins. *C*, N-terminal peptide sequences used for *in vitro* binding assays. *D*, schematic diagrams for direct interaction of LC8 dimers with N-terminal peptides in monomeric form or dimeric form. *E*, FSEC profiles showing elution of GFP proteins tagged by N-terminal peptides or LC8. Novel peaks (*arrowheads*), eluting earlier indicate interaction and consequent size enlargement. The protein composition of each sample is shown in SI SF 5. FSEC, fluorescence detection size-exclusion chromatography; Kir, inwardly rectifying potassium; LC8, light chain 8.

To identify the site of LC8 binding, we used LC8Pred (14), a web server that performs sequence-based predictions of LC8 binding site(s). This analysis identified a potential LC8 binding motif comprised of eight amino acids (9-YSQTTQTE-16) at the N-terminal loop of Kir4.1 (Fig. 3*A*) that includes the key phosphorylatable Tyr9 (8). The motif is absent in other Kir channels (Fig. 3*A*) but completely conserved between Kir4.1 orthologs (Fig. 3*B*), except in one species (vampire fish). The highly conserved anchoring sequence of three amino acids (13-Tyr-Gln-Tyr (TQT)-15) is crucial for LC8 interaction (14). To test the veracity of the predicted binding site, WT and ΔTQT (deletion of the three TQT residues) Kir4.1 N-term peptides (residues 1–38), as well as the strong LC8 binding motif of SPAG5 protein (14), were generated (Fig. 3*C*), and each of these peptides was tagged to the C terminus of either GFP or GST (Fig. 3*D*); the former forces the peptides to be monomeric, whereas the latter allows dimerization of the peptides. None of the GFP-tagged peptides showed a molecular weight shift after incubation with GST-LC8 (Fig. 3*E*). In contrast, GFP-LC8 eluted earlier after incubation with GST-SPAG5 or GST-WT peptides, indicating complex formation, but there was no shift with GST-ΔTQT peptide (Fig. 3*E*). The results indicate that the dimeric state of the peptides allowed binding to the Kir4.1 and SPAGS5 LC8-binding domains and that the TQT anchoring sequence is required for their interactions.

**Figure 4:**
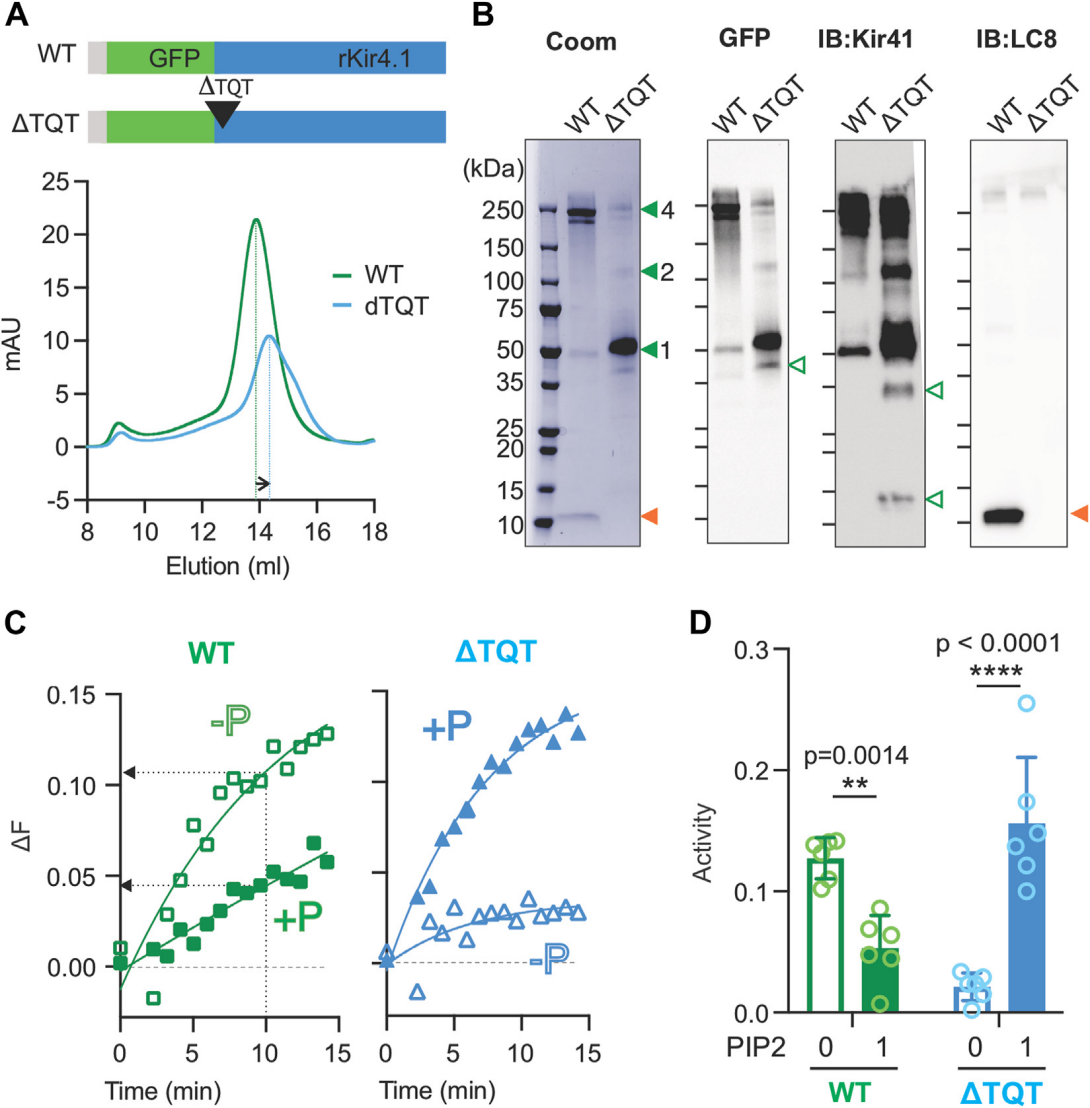
**Full-length Kir4.1 proteins with ΔTQT deletion mutation**. *A*, schematic diagram and SEC profiles (*below*) of WT and Kir4.1-ΔTQT constructs. Tetramer peaks are designated by *vertical dotted lines*, and *horizontal arrow* shows *right shift* of the Kir4.1-ΔTQT protein sample. *B*, The 1D gels were visualized by Coomassie staining, GFP signals, and anti-Kir4.1 or anti-LC8 antibodies. Tetrameric, dimeric, and monomeric bands (*filled green arrowheads*), truncated Kir4.1 proteins (*open green arrowheads*), and copurified LC8 (*orange arrowheads*) are indicated. Coomassie-stained membrane is shown in SI SF 6. *C*, fluorescence changes resulting from channel activity were measured using *in vitro* ACMA assay (15), conducted in the absence (-P) or presence (+P) of the agonist PIP_2_. *D*, relative fluorescence intensity changes at 10 min from experiments as in (*C*). n = 6 independent replicates, mean ± SD; *p* values were computed using two-way ANOVA with Sidak’s multiple comparison test (41). ACMA, 9-amino-6-chloro-2-methoxyacridine; Kir, inwardly rectifying potassium; LC8, light chain 8; PIP_2_, phosphoinositide-(4,5)-phosphate; SEC, size-exclusion chromatography.

To probe the role of the predicted binding site in LC8 interaction with the channel, full-length Kir4.1 protein with the ΔTQT deletion was overexpressed and purified (Fig. 4*A*). Kir4.1-ΔTQT purified as tetramers; the tetramer peak was broader and right-shifted, indicating that the mutant in solution is smaller than the WT (Fig. 4*A*). That this is due to loss of LC8 interaction (loss of 4 LC8 dimers would reduce the size by ∼80 kDa) is evident in Coomassie-stained gels and confirmed by immunoblot with anti-LC8 antibodies (Fig. 4*B*). The results demonstrate that the anchoring sequence is necessary for the interaction and hence that the predicted site is indeed a *bona fide* LC8 binding motif.

To probe the functional consequences of LC8 interaction, channel activity of purified WT and ΔTQT proteins was assessed using a liposome flux assay (15) (Fig. 4, *C* and *D*). The ΔTQT proteins exhibited normal behavior, wherein the channel was inactive but became activated by PIP_2_, strongly supporting that the ΔTQT proteins are properly folded. On the other hand, WT proteins exhibited abnormal behaviors, showing basal activity in the absence of PIP_2_ but becoming inhibited by PIP_2_, suggesting that the WT proteins potentially are in deformed conformations.

**Figure 5:**
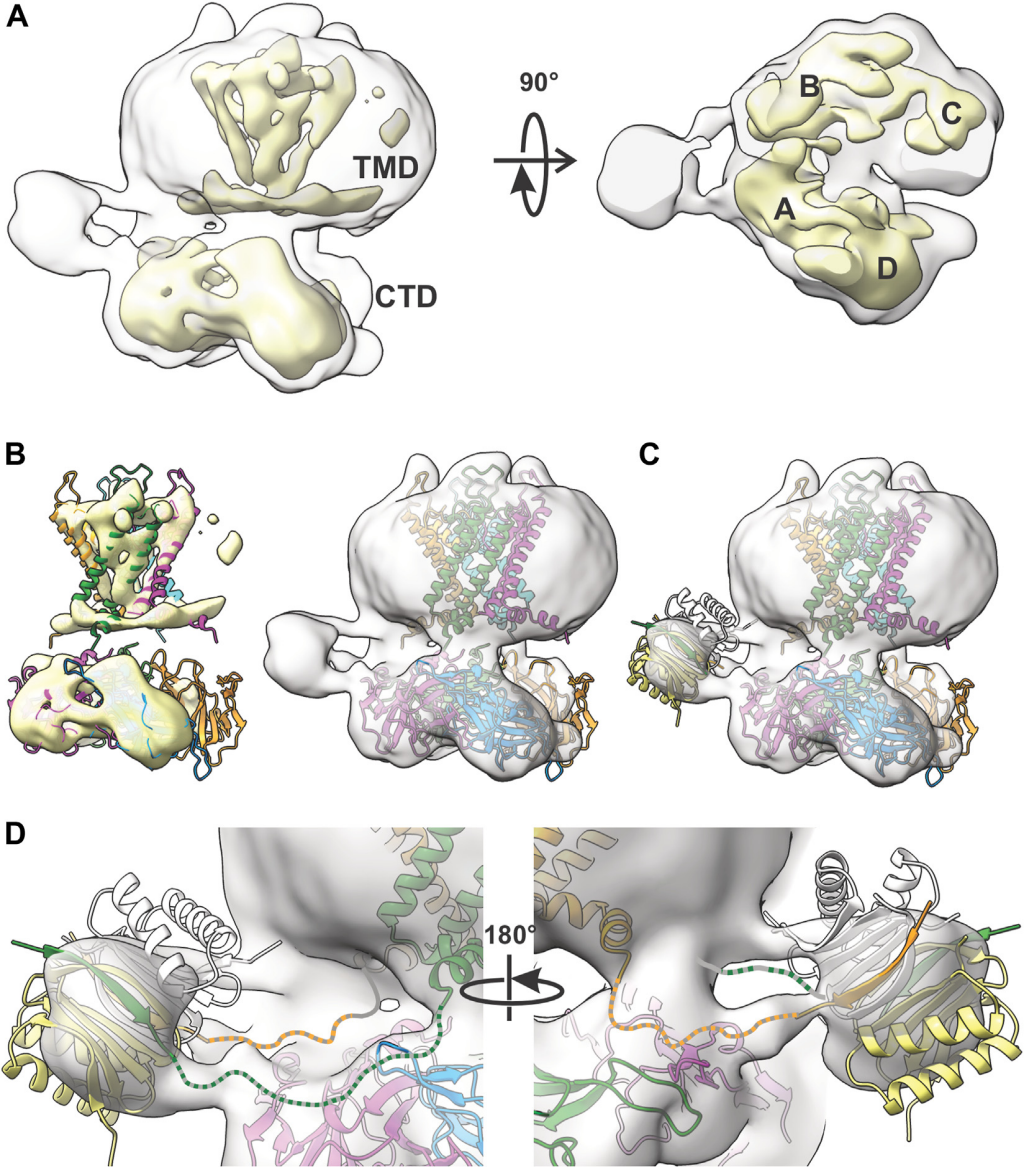
**Rigid body fit to cryo-EM map of full-length Kir4.1.***A*, electron density maps contoured at lower density level of 0.13 (*gray*, equivalent to sdLevel 2.9) or at higher density level of 0.34 (*yellow*, equivalent to sdLevel 7.6) viewed from the side (*left*) or from the *top* (*right*). In the latter, the TMD was omitted to show the CTD subunit densities. *B*, rigid body fit of each TMD and CTD subunit to the map contoured at high (*left*) or low (*right*) density. *C*, rigid body fit of an LC8 dimer to the auxiliary density on the side. *D*, zoom-in of the interface between the LC8 dimer and the Kir4.1 models. *Green* and *orange dashed lines* illustrate presumed conformation of the remaining N-terminal loops. CTD, cytoplasmic domain; Kir, inwardly rectifying potassium; LC8, light chain 8; TMD, transmembrane domain.

Single-particle cryo-EM was then attempted to determine the structural basis of LC8 interaction with WT Kir4.1. Despite the monodispersed tetramer peak observed with size-exclusion chromatography (SEC, Fig. 1*C*) and a large number of particles (over 900,000), particles were highly heterogeneous, particularly in the cytoplasmic domain (SI SF 7). At the achieved resolution (6.7 Å), it is clear that fourfold symmetry is lost in the cytoplasmic region, with one additional density associated with each tetramer, and located at the crevice between the transmembrane domain (TMD) and the cytoplasmic domain of one pair of subunits (SI SF 7 and Fig. 5*A*). The size of the additional density is comparable to the LC8 dimer density seen in dynein complexes (16) when both maps are contoured at the same density level (SI SF 8). Although the available resolution precludes formal model building, rigid body fitting of an LC8 dimer (Protein Data Bank code: 3E2B) (17), guided by continuous densities connecting the putative LC8 density to the Kir4.1 protein density (Fig. 5*B*), positions the dimer such that the Kir4.1 N-terminal LC8 binding motifs, now adopting a β-strand (Fig. 5, *C* and *D*, *left*, *green* and *right*, *orange*), point toward Kir4.1. The continuous densities suggest that the LC8 dimer forms an interaction with two neighboring Kir4.1 subunits. The interaction pulls the two interacting subunits closer to the TMD and into tight engagement with each other but conversely causes the other two subunits to disengage from the TMD and from each other (Fig. 5*A*), potentially underlying the abnormal channel activity of the purified WT proteins.

## Discussion

In this study, we have identified DYNLL1, and the paralog dynein light chain 2, collectively referred to as LC8 proteins, as directly interacting with the unstructured Kir4.1 N-terminal loop. Interaction between Kir4.1 and LC8 proteins is supported by a high-throughput affinity-purification mass spectrometry study (18) and can be searched from the BioGRID web server (19).

LC8 dimers were first discovered as component of cytoplasmic dynein complexes but later found to independently interact with many other proteins that contain LC8 binding motif(s) (14). Among ion channels, VDAC (20) and Kv7.4 (21) have recently been reported to interact with, and be controlled by LC8, although neither of these channel proteins generate positive LC8Pred server hits for LC8 binding, and so whether regulation is by direct binding to LC8 is unclear. On the other hand, direct binding of another dynein light chain dimer (Tctex-1) to G-protein–coupled receptors (22, 23, 24) and N-/P-type Ca^2+^ channels (25) has been shown to regulate surface expression, and it is possible that LC8 interactions function in such a manner to control Kir4.1 surface expression. Kir4.1 surface expression is augmented by phosphorylation of Tyr9 in the LC8 binding motif (8), and conceivably, the negative charged phosphate moiety may interfere with LC8 interaction, in parallel with the negative effect of Ser phosphorylation on LC8 interaction with Nek9 (26). By reducing dynein complex–driven retrograde movement of Kir4.1 channels from the plasma membrane, such reduced LC8 interaction may thereby facilitate dynamic regulation of Kir4.1 surface expression (27, 28).

Our results suggest that LC8 interaction may also have direct effects on Kir4.1 protein function. First, loss of LC8 interaction made the tetrameric assembly less stable (Fig. 4*B*). While Kir4.1-WT proteins mainly stay as tetramers, Kir4.1-ΔTQT proteins disassemble and mainly run as monomers during 1D SDS-PAGE, indicating that LC8 interaction may be important for tetramer stability. Second, loss of LC8 interaction made the protein more susceptible to degradation. Additional bands smaller than the monomer band, at around 50 kDa, in the Kir4.1-ΔTQT sample were essentially absent in the WT sample. Third, LC8 interaction had a direct effect on channel activity *per se*. While ΔTQT proteins, which lack the capability to interact with LC8, exhibited normal channel activity, with marked activation by PIP_2_, both structural and *in vitro* functional data suggest that purified Kir4.1 WT proteins are in a relatively defective state and lack PIP_2_ activation, The EM density indicates that interaction of one LC8 dimer with two neighboring WT Kir4.1 subunits leads to a marked loss of fourfold symmetry. Future studies are required to address the extent and role of LC8 interaction with Kir4.1 within cells, but the present study makes it clear that LC8 does directly bind to Kir4.1 and can have significant regulatory effects.

## Experimental procedures

### Cloning and stable cell line generation

N-terminal FLAG tag and TQT deletions were introduced to GFP-tagged Kir4.1 complementary DNA in p-EGFP vector (29) by high-fidelity PCR and then subcloned into the attB recombination plasmid (30). Constructs were stably transfected into human embryonic kidney 293 cells using the Landing Pad system (30) and selected through multiple rounds of subculturing in the presence of 10 μM puromycin or 10 nM Rimiducid (MedChemExpress) that activates inducible caspase-9 (31). Stable cells in adherent mode were then converted to suspension cultures by switching to serum-free medium (32). Suspension cultures were started at around 0.5 × 10^6^ cells/ml and cultured at 145 rpm and 37 °C in 8% CO_2_ and then subcultured at cell density ∼3 × 10 (6) cells/ml.

### Overexpression and purification

Details are provided in the Supporting Information. Briefly, stably transfected cells in suspension were induced by doxycycline for 3 days and then harvested. Cells were lysed by sonication, and membrane solubilization was achieved by 1% lauryl maltose neopentyl glycol and 0.12% cholesterol hemisuccinate. Affinity purification was performed using FLAG resin (Sigma; catalog no.: A2220) with exchange to a glyco-diosgenin containing buffer during the washing step. Proteins were eluted by overnight cleavage by human rhinovirus 3C proteases and further purified by SEC using a Superose 6 column in glyco-diosgenin-containing buffer. Tetramer fractions were collected and concentrated to approximately 7 to 8 mg/ml for peptide mass spectrometry and vitrification.

### Peptide mass spectrometry

Details are provided in the Supporting Information. Briefly, peptides were prepared using a modified filter-aided sample preparation method (33). Samples from the top two Kir4.1 bands and the ∼10 kDa band were prepared using in-gel digestion (34) with minor modifications. Samples were analyzed using ultra–high performance mass spectrometry (35) using a hybrid quadrupole Orbitrap LC-MS System, Q-Exactive PLUS interfaced to an EASY-nanoLC 1000, and MS2 spectra were analyzed using Mascot software (Matrix Science; version 2.5.1) (36). Each of these experiments was performed once, and the raw and processed data are deposited to the ProteomeXchange Consortium *via* the PRIDE (37) partner repository with the dataset identifier PXD060340 and provided as Supporting Information.

### *In vitro* pulldown assay

Details are provided in the Supporting Information. Briefly, GST-conjugated LC8 (GST-LC8) clone was obtained from Addgene (13), and the overexpressed proteins in *Escherichia coli* were purified through one-step affinity purification with glutathione resins (Cytiva; catalog no.: 17075605). GST and LC8 were separated by human rhinovirus 3C protease overnight and separately purified. PBS was used in all steps. Pulldown of Kir4.1 proteins was visualized by GFP signals or immunoblotting with anti-FLAG antibodies (Invitrogen; catalog no.: MA1-918878-HRP) or anti-Kir4.1 antibodies (Alomone Lab; catalog no.: APC-035).

### *In vitro* peptide binding assay

GFP- or GST-tagged N-terminal peptides as well as LC8 proteins were overexpressed in *E. coli* BL21 and purified with nickel–nitrilotriacetic acid resins (Thermo Scientific; catalog no.: 88222) *via* N-terminal 6xHis tag and subsequent SEC (Superdex 200) equilibrated with PBS. Fluorescence SEC (Shimadzu) through a Superdex 200 column was used to detect the size change of GFP-tagged N-terminal peptides or LC8 proteins through interaction with GST-tagged LC8 or N-terminal peptides, respectively.

### *In vitro* 9-amino-6-chloro-2-methoxyacridine flux assay

Details are provided in the Supporting Information. Briefly, purified proteins were reconstituted in synthetic liposomes composed of POPE:POPG in an 8:2 ratio, with or without 1% (w/w) brain PIP_2_. The activity of the protein was inferred from the fluorescence change of 9-amino-6-chloro-2-methoxyacridine; a greater fluorescence change indicates higher activity.

### Single-particle cryo-EM

Details for single-particle EM imaging and analysis can be found in the Supporting Information. Briefly, the grids were prepared using FEI Vitrobot Mark IV with purified Kir4.1 supplemented with diC8 PIP_2_. Movies were recorded with a pixel size of 0.657 Å and analyzed using CryoSPARC (Structura Biotechnology Inc), version 4.5.3. Three (blob and template-based picking and Topaz picking) approaches were employed to detect single particles from motion-corrected and contrast transfer function–estimated micrographs, identifying more than 900,000 particles. From these, approximately 300,000 particles isolated from heterogeneous refinement resulted in the best electron density map at a resolution of 6.69 Å, which was deposited in the Electron Microscopy Data Bank (EMD-48154). Further 3D classification of the 300,000 particles identified a subgroup (149,671 particles) with additional density at the crevice. Nonuniform refinement of this subgroup yielded a map with lower global resolution of 6.93 Å but with finer details of the additional density, to which a rigid body fit was manually applied, as shown in Figure 5. Protein coordinates are provided as Supporting Information (Kir4.1_LC8_complex.pdb). Structural illustrations were prepared using UCSF ChimeraX-1.8 (38).

### Multiple sequence alignment

Multiple sequence alignments were made using the Clustal Omega web server (39), and the sequence logo was generated by WebLogo server, version 2.8.2 (40).

### Statistical analysis

Statistical significance was analyzed using two-way ANOVA. Statistical significance of *p* < 0.05, *p* < 0.01, and *p* < 0.001 is indicated by single, double, and triple asterisks, respectively.

## Data availability

The authors declare that the data underlying the findings of this study are available within the article and its Supporting Information and are available upon request.

## Supporting information

This article contains supporting information (13, 17, 33, 35, 36, 37, 42, 43, 44, 45, 46, 47, 48).

## Conflict of interest

The authors declare that they have no conflicts of interest with the contents of this article.

## Supporting information

SupportingInformation_Figures

SupportingInformation_Method

SupportingInformation_ST1

SupportingInformation_Table

Kir4.1_LC8_complex
