# Supplementary material for: Dynein light chains 1 and 2 are auxiliary proteins of pH-sensitive Kir4.1 channels
Source: J Biol Chem. 2025 Mar 10;301(4):108393. doi: 10.1016/j.jbc.2025.108393 (PMC11999606; doi:10.1016/j.jbc.2025.108393)
Supplement: SupportingInformation_Figures [file mmc1.docx]

**Dynein light chains 1 and 2 are auxiliary proteins of pH-sensitive Kir4.1 channels**

# Sun-Joo Lee^1,#^, Jian Gao^1^, Ellen Thompson^1^, Jonathan Mount^2^, and Colin G. Nichols^1^

^1^Department of Cell Biology and Physiology and the Center for Investigation of Membrane Excitability Diseases, Washington University School of Medicine, St. Louis, Missouri, USA,

^2^Department of Anesthesiology, Weill Cornell Medical College, New York, NY, USA

^#^ To whom correspondence should be addressed.


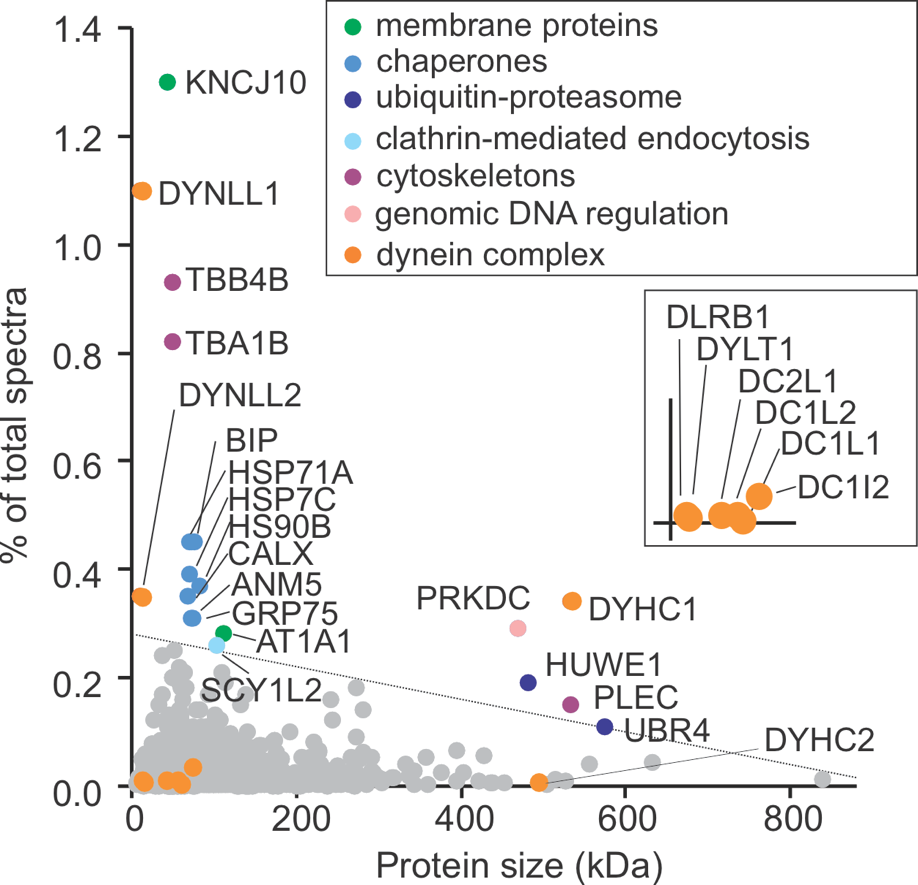


**Supporting Information Figure SF1. Kir4.1 interactome of HEK293 cells.** The proteins that co-purified with overexpressed Kir4.1 in HEK293 stable cells were identified using the peptide mass fingerprinting method. These proteins are plotted by their size and abundance (percentage of total spectra) in the Kir4.1 solution sample. Only the proteins with the high abundance are named and color-coded based on their cellular activities. The other components of cytosolic dynein complexes are additionally marked in orange, and the lower abundance proteins among them are zoomed in in the inset. The full list of the co-purified proteins is provided in Supporting Information ST1.


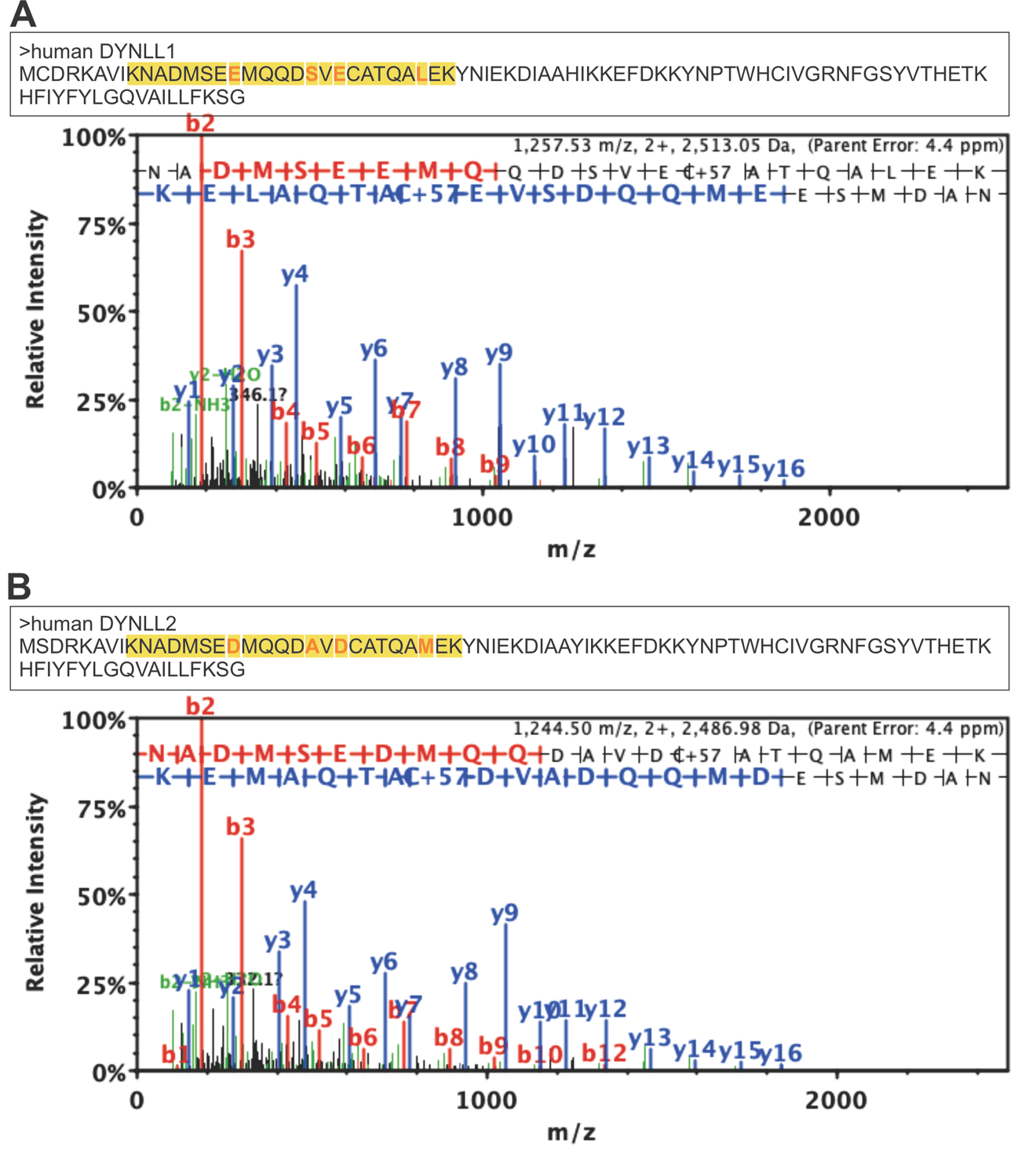


**Supporting Information Figure SF2. Exemplary unique MS/MS spectra of human dynein light chain 1 (A) and 2 (B)** The protein sequences of each protein are shown in the boxes, with the representative and unique segments corresponding to the presented spectra highlighted in yellow. The different amino acids between the two proteins are marked in red.


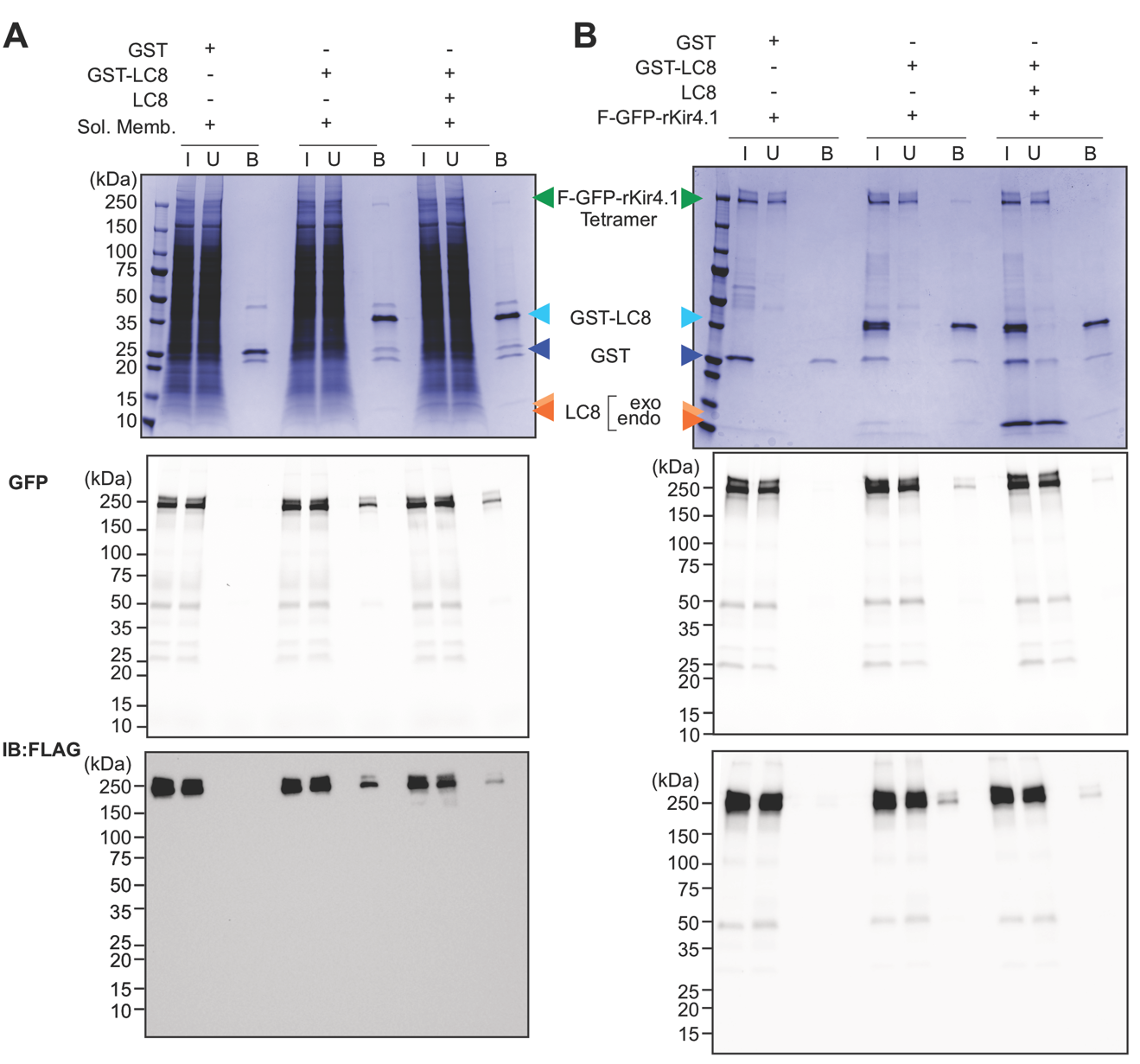


**Supporting Information Figure SF3. Whole gel images for the LC8 pull down assays**

The full gel images of Fig. 2B are shown. The major bands are marked by arrowheads and the corresponding protein names.


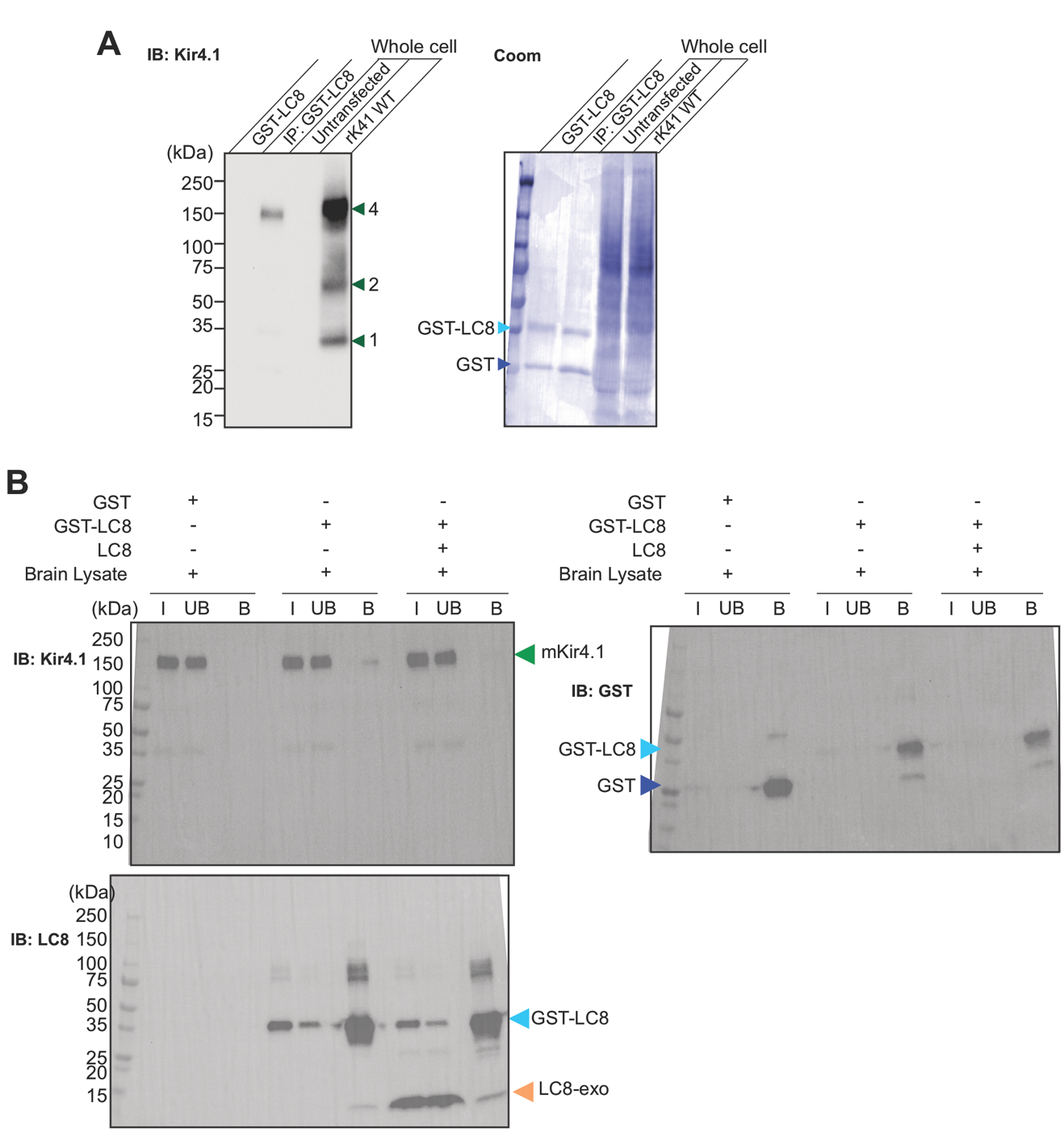


**Supporting Information Figure SF4. Whole gel images for the LC8 pull down of native Kir4.1 proteins. A)** Specific detection of Kir4.1 by anti-kir4.1 rabbit polyclonal Abs. Immunoblotting image (Left) and the Coomassie stained membrane image (Right) are shown. LN1: GST-LC8; LN2: mouse brain proteins pulled down with GST-LC8; LN3: the whole lysate of un-transfected HEK293 cells; LN4: the whole lysate of rKir4.1 wild type without any tags transfected cells. Kir4.1 bands observed via immunoblotting are marked by arrowheads and the numbers indicating expected oligomeric state of the proteins. **B)** The full gel images of Fig. 2C are shown. The major bands are marked by arrowheads and the corresponding protein names.


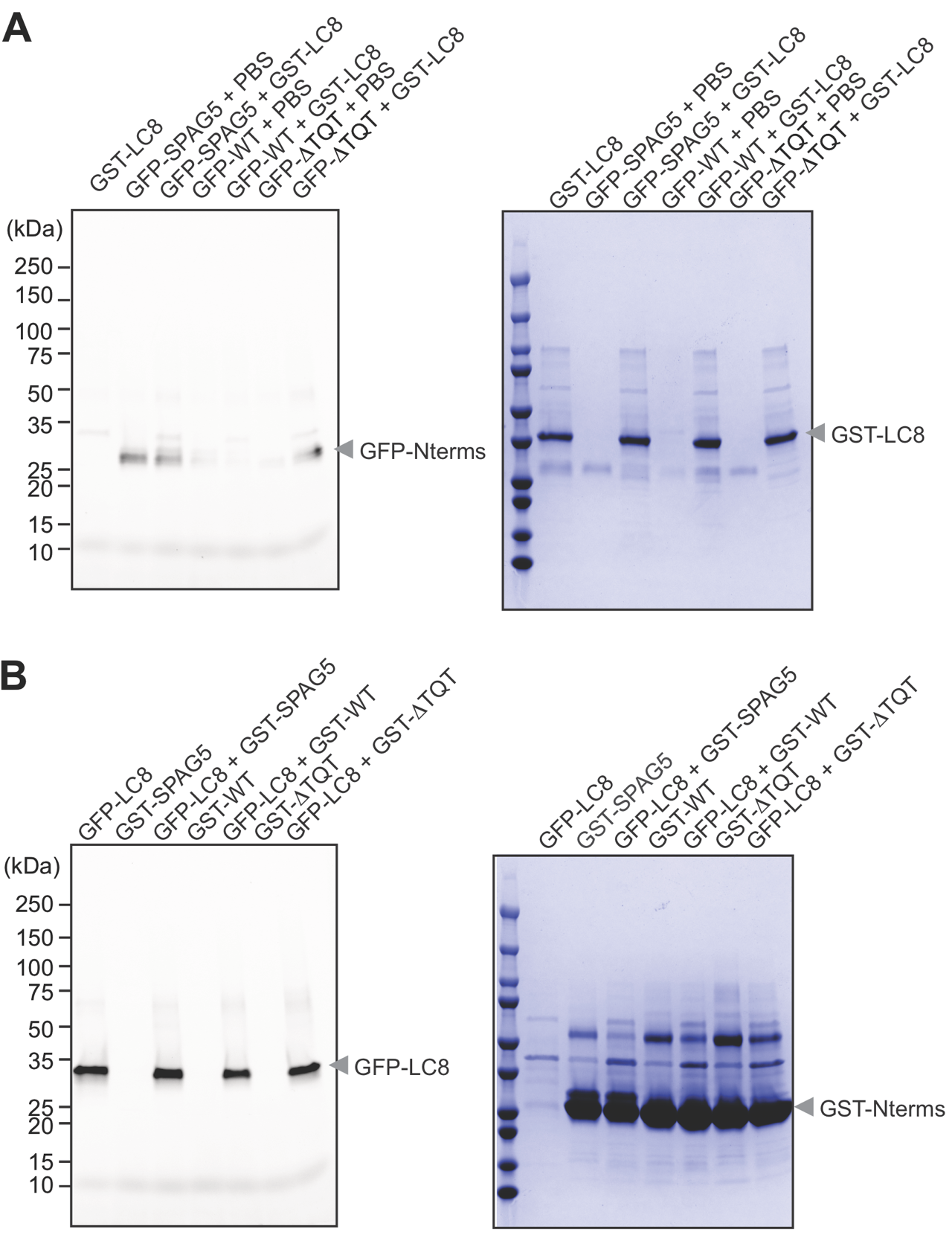


**Supporting Information Figure SF5. 1D profiles of the protein sample mixes used for the in vitro peptide binding assay via FSEC.** Protein samples of the experiments shown in Fig. 3E Left **(A)** or Fig. 3E Right **(B)**. The 1D gel was imaged for GFP signals (Left) first and then visualized by Coomassie staining (R).

**
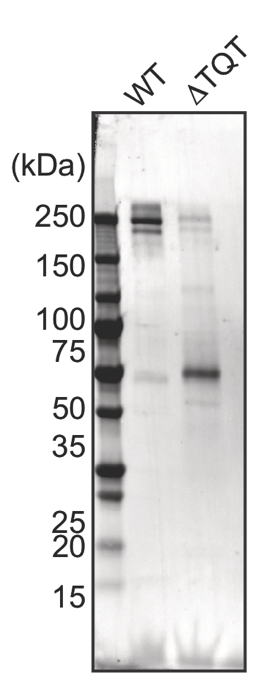
**

**Supporting Information Figure SF6. Coomassie stained membrane** of Fig. 4B showing that an equal amount of purified Kir4.1-WT and Kir4.1-ΔTQT proteins was loaded for the immunoblotting.

**
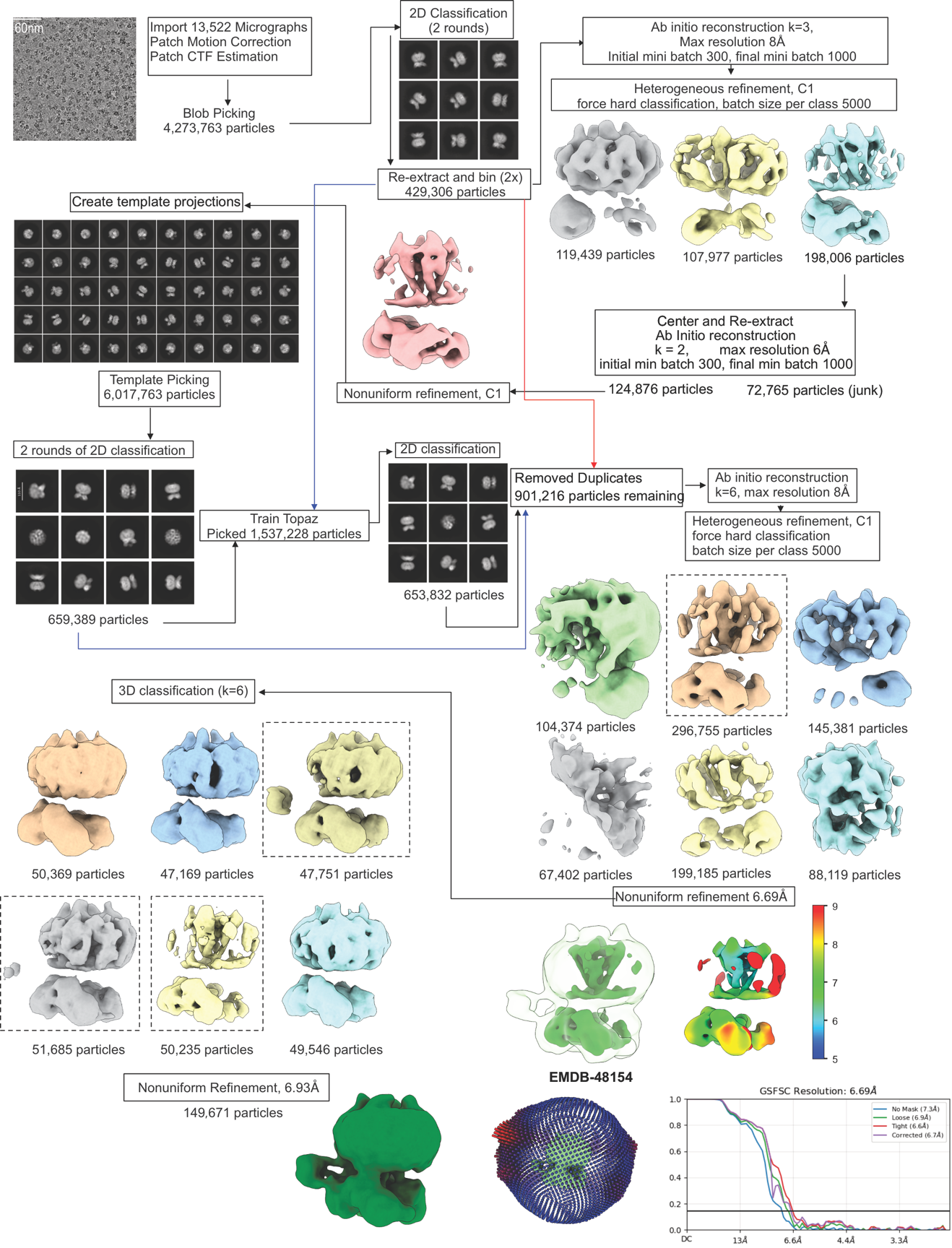
**

**Supporting Information Figure SF7. Flow chart of single particle cryo-EM analysis**

**
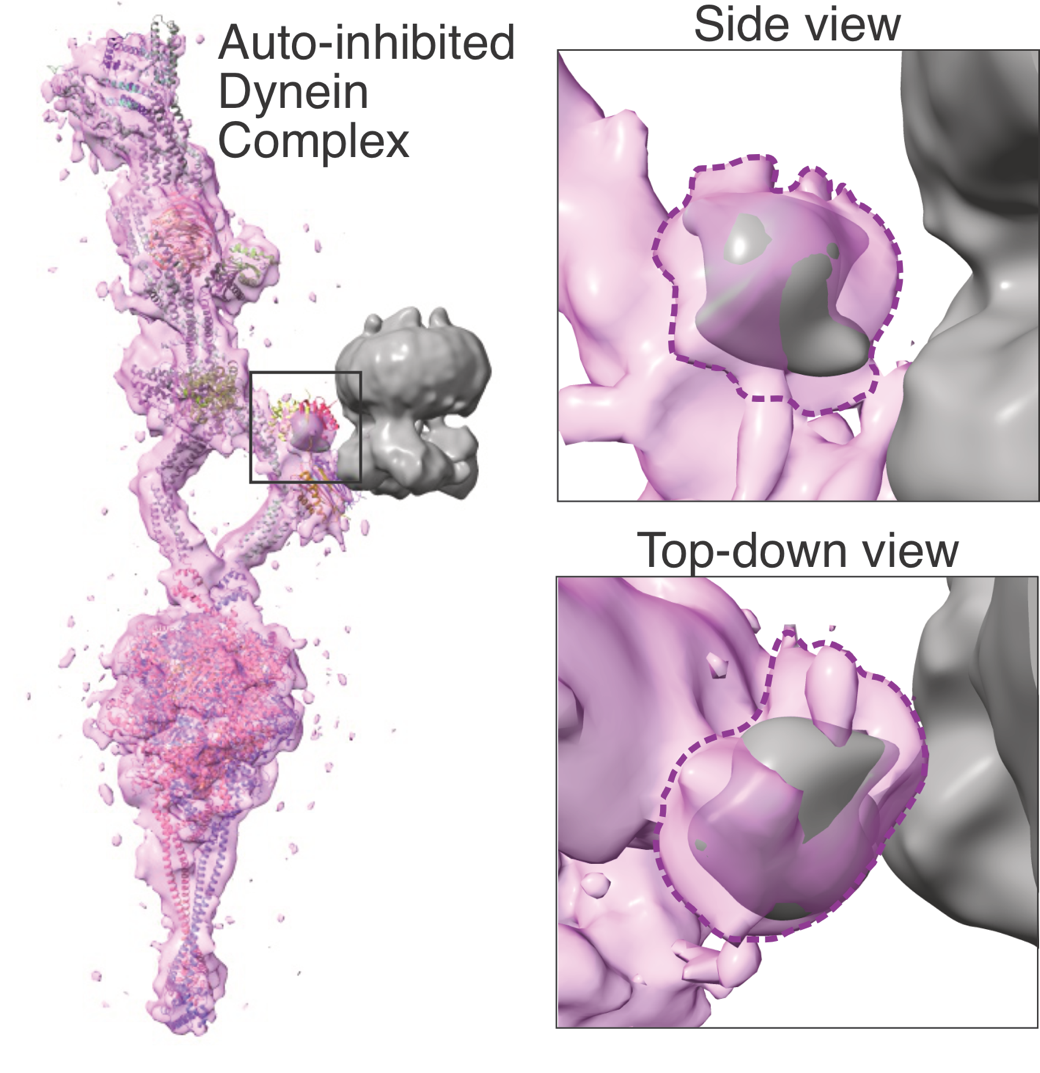
**

**Supporting Information Figure SF8. Coomassie stained membrane**

Electron density (EMD-3705:15 Å) and structural model (PDB:5NVU) of cytoplasmic dynein complex in the auto-inhibited state are shown in pink. The auxiliary density of the full length Kir4.1 (gray) is overlayed on the LC8 density marked by magenta dashed line. The two electron density maps are contoured at the same level.
