# Supplementary material for: Dynein light chains 1 and 2 are auxiliary proteins of pH-sensitive Kir4.1 channels
Source: J Biol Chem. 2025 Mar 10;301(4):108393. doi: 10.1016/j.jbc.2025.108393 (PMC11999606; doi:10.1016/j.jbc.2025.108393)
Supplement: SupportingInformation_Method [file mmc2.docx]

**Dynein light chains 1 and 2 are auxiliary proteins of pH-sensitive Kir4.1 channels**

# Sun-Joo Lee^1,#^, Jian Gao^1^, Ellen Thompson^1^, Jonathan Mount^2^, and Colin G. Nichols^1^

^1^Department of Cell Biology and Physiology and the Center for Investigation of Membrane Excitability Diseases, Washington University School of Medicine, St. Louis, Missouri, USA,

^2^Department of Anesthesiology, Weill Cornell Medical College, New York, NY, USA

^#^ To whom correspondence should be addressed.

**Detailed Materials and Method*s***

***Overexpression and purification of full-length Kir4.1***

800 mL cultures were seeded at ~1.5x10^6^ cells/mL for one day and replenished with 0.25 % Primatone, 0.2 % glucose, and 2 µM doxycycline for induction. After 12-hour incubation at 37 °C, cultures were treated with 5 mM butyrate to stop cell division and continued for another 2.5 days at 30 °C. Cells were harvested by centrifugation at 200g for 10 min at 4 °C, washed in ice-cold PBS, and transferred to 50 mL tubes. Cell pellets were kept frozen at -80 °C until purification. Cell pellets were thawed at 4 °C and sonicated in lysis buffer supplemented with DNaseI, 2 β-mercaptoethanol (2-ME) and protease inhibitors including 0.1 mg/mL AEBSF, 0.1 μg/mL Pepstatin A, 1 μg/mL Leupeptin, 1 μg/mL Apotrotinin, and 1 PMSF. Large debris were removed by 5 min 400 xg centrifugation and subsequent centrifugation for 35 min at 2,500g, all at 4 °C. Membrane fractions were collected by centrifugation of the supernatant at 100Kg for 2 hours and resuspended in solubilization buffer (in mM, 50 Tris pH 8.0, 150 KCl, 2 2-ME, 1 EDTA with 1% Lauryl maltose neopentyl glycol (LMNG), 0.12% cholesterol hemisuccinate (CHS)). After 3-hour solubilization, insoluble fractions were removed by centrifugation at 30Kg for 30 min. Supernatants were incubated with 0.5 mL FLAG resin (Sigma, A2220) per 1.6 L culture for 2 hours, and the resin was washed with 20x resin volume of washing buffer (in mM, 50 Tris pH 8.0, 150 KCl, 2 2-ME, 1 EDTA, 0.085 Glyco-diosgenin (GDN)). Proteins were eluted by overnight cleavage by HRV C3 proteases. Eluted samples were further purified via a Superose 6 column equilibrated with size exclusion buffer (in mM, 20 Tris pH8.0 (or 6.0), 150 KCl, 1 TCEP, 1 EDTA, 0.04 GDN). [KCl] was increased to 500 mM in all buffers when higher ionic strength was used. Tetramer fractions were collected and concentrated to ~8 mg/mL for peptide mass spectrometry and vitrification.

For pulldown assays and for in vitro functional assays, reducing reagents were omitted in all purification steps. Cell lysis was performed by homogenization in hypotonic buffer (10 Tris pH8.0, 15 KCl, 2 EDTA) with protease inhibitors and DNaseI. Lysates were subjected to membrane fractionation by 1 hour ultracentrifugation at 100Kg, and pellet were resuspended in solubilization buffer for 3 hours. After insoluble fractions were removed by centrifugation, supernatants were used directly for pulldown assay, or were subject to affinity and SEC to obtain purified Kir4.1 proteins. In this case, proteins were eluted by 500 ug/mL FLAG peptide (Sigma, F3290) to keep the N-terminal GFP.

***Peptide mass spectrometry***

*Solution sample digestion*

Peptides were prepared from the rKir_41 solution sample using a modified filter-aided sample preparation (FASP) method^1^. The sample was mixed with 200 µl of 100 mM Tris-HCL buffer, pH 8.5 containing 8 M urea (Sigma, Cat. No. U4884) (UA buffer). Protein disulfide bonds were reduced using 20 mM dithiothreitol (Pierce, Cat. No. A39255) (DTT) with heating to 37 ºC for 1 h. The sample was transferred to the top chamber of a 10,000 molecular weight cut-off (MWCO) filtration unit (Millipore, Cat. No. MRCF0R010) and spun in a microcentrifuge (Eppendorf) at 14,000 rcf for 10 min. An additional 200 µl of UA buffer was added and the filter unit was spun at 14,000 rcf for 15 to 20 min. The cysteine residues were alkylated using 100 µl of 50 mM Iodoacetamide (IAM) (Pierce, Cat. No. A39271) in UA buffer. IAM in UA buffer was added to the top chamber of the filtration unit.

The sample was gyrated at 550 rpm for 30 min in the dark at RT using a Thermomixer (Eppendorf). The filter was spun at 14,000 rcf for 15 min and the flow through discarded. Unreacted IAM was washed through the filter with two sequential additions of 200 µl of UA buffer, and centrifugation at 14,000 rcf for 15 to 20 min after each buffer addition. The flow through was discarded after each buffer exchange- centrifugation cycle. The UA buffer was exchanged with digestion buffer (DB), 50 mM ammonium bicarbonate buffer, pH 8. Two sequential additions of DB (200 µl) with centrifugation after each addition to the top chamber was performed. The top filter units were transferred to a new collection tube and 100 µl DB containing 1 mAU of LysC (Wako Chemicals, cat. no. 129-02541) was added and sample was incubated at 37 °C for 2 h. Trypsin (1 µg) (Promega, Cat. No. V5113) was added and sample was incubated overnight at 37 °C. The filter was spun at 14,000 rcf for 15 min to recover the peptides in the lower chamber. The filter was washed with 50 µl of DB buffer and the wash was combined with the peptides. The peptides were acidified to 1% (vol/vol) TFA in preparation for desalting using stage tips (C18) as previously described^2^. The peptides were eluted with 60 µl of 60% (vol/vol) Acetonitrile (J.T. Baker) (MeCN) in 0.1% (vol/vol) formic acid (Fluka) (FA) and dried in a Speed-Vac (Thermo Scientific, Model No. Savant DNA 120 concentrator). The peptides were dissolved in 20 µl of 2% (vol/vol) MeCN in water. An aliquot (10%) was removed for quantification using the Pierce™ Quantitative Fluorometric Peptide Assay kit (Thermo Scientific, Cat. No. 23290). The remaining peptides were transferred to an autosampler vial (Sun-Sri, Cat. No. 200046), dried and stored at -80 °C.

*In-gel digestion*

Gel plugs were digested as previously described^2^ with minor modifications. Briefly, three 1mm diameter cores were cut from each Comassie stained gel band and transferred to separate wells in a 96 well PCR microplate (Axygen, Cat. No. PCR-96M2-HS-C). Gel plugs were de-stained in 100 µL of 50 mM ammonium bicarbonate (ABC) buffer containing 50% MeCN. Gel plugs were dehydrated by two incubations for 15 min at room temperature in 100% MeCN. Dehydrated plugs were reduced in 100 µL 10 mM DTT at 56 °C for 30 minutes. Excess reagent was aspirated, and plugs were alkylated with 100 µl of 100 mM ABC buffer containing 55 mM iodoacetamide for 20 min in the dark at room temperature. Excess reagent was aspirated, and plugs were dehydrated with 100% MeCN. Trypsin digestion was initiated by addition of trypsin in 50 mM ABC buffer containing 10% (vol/vol) MeCN. Enough volume was added to cover the dehydrated plug. Digestion was carried out for 30 min at 37 °C. A second aliquot of trypsin was added for overnight digestion at 37 °C. The next morning, peptides were extracted from the gel in 50% (vol/vol) MeCN in aqueous 5% (vol/vol) FA for 20 min at 37°C. Extraction buffer was aspirated and transferred to an autosampler vial. Extraction step was repeated once, and extraction buffer was combined with the first aspirate into the same autosampler vial. Peptides were dried in a speedVac and stored at -80 °C until LC-MS analysis

*Mass spectrometry*

The samples were analyzed using ultra-high performance mass spectrometry^3^ using a hybrid quadrupole Orbitrap LC-MS System, Q-Exactive™ PLUS interfaced to an EASY-nanoLC 1000. A 75 µm i.d. x 50 cm Acclaim PepMap 100 C18 RSLC column (Thermo Scientific™) was equilibrated with 100% solvent A (1%FA) on the nano-LC for a total of 11 μl at 700 bar pressure. Samples in FA 1% (vol/vol) were loaded at a constant pressure of 700 bar. Peptide chromatography was initiated with mobile phase A (1% FA) containing 2% solvent B (100%ACN, 1%FA) for 5 min, then increased to 20% B over 100 min, to 32% B over 20 min, to 95% B over 1 min and held at 95% B for 19 min, with a flow rate of 250 nl/min. Data were acquired in data-dependent mode. Full-scan mass spectra were acquired with the Orbitrap mass analyzer using a scan range of *m/z* = 325 to 1500 and a mass resolving power set to 70,000. Ten data-dependent high-energy collisional dissociations were performed with a mass resolving power set to 17,500, a fixed lower value of *m/z* 100, an isolation width of 2 Da, and a normalized collision energy setting of 27. The maximum injection time was 60 ms for parent-ion analysis and product-ion analysis. The target ions that were selected for MS/MS were dynamically excluded for 15 sec. The automatic gain control (AGC) was set at a target value of 1e6 ions for full MS scans and 1e5 ions for MS2. Peptide ions with charge states of one or > 8 were excluded for HCD acquisition.

The MS2 spectra from peptides with +2, +3 and +4 charge states were analyzed using Mascot software^4^ (Matrix Science, London, UK; version 2.5.1). Mascot was set up to search against a UniProt database of human proteins (ver July 2019; 20,667 entries) assuming the digestion enzyme was trypsin with a maximum of 4 missed cleavages allowed. The searches were performed with a fragment ion mass tolerance of 20 ppm and a parent ion tolerance of 20 ppm. Carbamidomethylation of cysteine was specified in Mascot as a fixed modification. Deamidation of asparagine, deamidation of glutamine, acetylation of protein N-terminus, pyro-glutamate formation from n-terminal glutamine residues and oxidation of methionine were specified as variable modifications. Peptides were filtered at 1% false-discovery rate (FDR) by searching against a reversed protein sequence database and protein identification requires observation of a minimum of 2 peptides.

The mass spectrometry proteomics data have been deposited to the ProteomeXchange Consortium via the PRIDE^5^ partner repository with the dataset identifier PXD060340 and 10.6019/PXD060340.

***In vitro pulldown assay***

For the pulldown assays, GST-conjugated LC8 (GST-LC8) was obtained from Addgene^6^, expressed in *E. coli* and purified through one-step affinity purification with glutathione resins (Cytiva, 17075605). GST and LC8 were separated by HRV 3C protease treatment overnight. Free LC8 was obtained by incubation with Ni-NTA resin (Thermo Scientific, 88222), which retains free GST containing N-terminal 6X His tag. Free GST was isolated from the fraction bound to glutathione resin among the eluates from Ni-NTA resins. Phosphate buffered saline (PBS) was used in all purification steps. Pulldown of Kir4.1 proteins from detergent-solubilized membrane fractions or from purified proteins was performed by incubation with either free GST or GST-LC8. Free LC8 was added to compete against GST-LC8 interaction with Kir4.1. Kir4.1 proteins were visualized by GFP signals or immunoblotting with anti-FLAG Abs (Invitrogen, MA1-918878-HRP) or anti-Kir4.1 Abs (Alomone Lab, APC-035). GST, GST-LC8, and both native (endo) and recombinant (exo) LC8 proteins were visualized by Coomassie staining or immunoblotting with anti-GST Abs (Santa Cruz Biotechnology, sc-9996) or anti-LC8 Abs (Invitrogen, PA5-65251). Anti-Na+/K+ ATPase 1 Abs (Cell Signaling, 3010S) and Anti-SWELL1 Abs (Custom made)^7^ were used for immunoblotting. All the commercially available antibodies were thoroughly tested and confirmed by each company, and anti-SWELL1 Ab has been characterized in the previous study^7,8^.

***In vitro ACMA flux assay***

1-palmitoyl-2-oleoyl-sn-glycero-3-phosphoethanolamine (POPE) and 1-palmitoyl-2-oleoyl-sn-glycero-3-phosphoglycerol (POPG) stocks were prepared at 10 mg/mL concentration in 20 mM Hepes, pH 7.5, 150 mM KCl, 20 mM CHAPS, 0.5 mM EGTA. They were mixed with lipids at 20 % (w/w) POPG and 80 % POPE with or without 1 % brain PI(4,5)P_2_ (PIP2) in 100 µL volume, and the mix was supplemented with 2 mM TCEP to keep the lipids and proteins from being oxidized^9^. Then 3 **μ**g of protein was added to each 100 µL lipid-detergent mix, giving 1:300=Protein:Lipid mass ratio. After incubation for 3 hours at RT, the protein-lipid-detergent mix was loaded on the dehydrated Sephadex G50 column that was equilibrated with the internal buffer (20 mM Hepes pH 7.5, 150 mM KCl, 0.5 mM EGTA buffer). 10 µL of proteo-liposome was added to each well of a 96-well plate, and then 190 µL of the flux buffer containing 2 µM 9-Amino-6-Chloro-2-Methoxyacridine (ACMA) in 20 mM Hepes pH 7.5, 150 mM NMDG, 0.5 mM EGTA buffer was added to get the initial base fluorescence intensity by Cytation 5 (BioTek, CA, USA) with excitation at 420 ± 20 nm and emission at 490 ± 20 nm. After 3-5 min, the proton ionophore Carbonyl Cyanide 3-ChloroPhenylhydrazone (CCCP) was added to trigger proton influx down the electrical gradient generated as a result of K^+^ efflux through active channel proteins and the ACMA quenching in an activity-dependent manner. After 12 mins of recording, valinomycin was added to trigger the maximum fluorescence decay for normalization.

# The fluorescence decay due to channel activity was calculated by subtracting the minimum fluorescence intensity from all readings and then normalizing all reads by the basal fluorescence intensity before addition of CCCP. The channel activity was inferred from the differential fluorescence levels (∆F) between the empty liposomes (Ø) with no added proteins and proteoliposomes at 10 min after CCCP treatment (Fig. 4D).

***Single particle cryo electron microscopy***

Cryo-EM grids were prepared using FEI Vitrobot Mark IV (FEI). 3 μl of purified Kir4.1 protein with N-terminal Flag-GFP tag cleaved off (~7 mg/mL supplemented with 10x higher mole fraction of D-myo-Phosphatidylinositol 4,5-bisphosphate (PI(4,5)P_2_ diC8), Avanti Polar Lipids 850185) was applied. Samples were applied to glow-discharged Cu Quantifoil R2/2 holey carbon grids. Grids were blotted for 2 s at 100% humidity and flash frozen in liquid ethane, then loaded onto a Krios (FEI) electron microscope operating at 300 kV equipped with a Falcon 4 (FEI) detector. Movies were recorded using the EPU software with a pixel size of 0.657 Å and a nominal defocus value between -0.8 to -2.4 μm. Data were collected with a dose of ~ 11.32 electrons per Å^2^ per sec, and each movie was recorded with 49 frames (87.55 ms per frame) for a 4.28 sec exposure.

**Single particle analysis**

Single particle analysis was performed using Cryosparc4.5.3^10^. Patch motion correction and subsequent CTF estimation were carried out on 13,522 micrographs obtained in super-resolution mode (0.657 Å/pixel). Auto picked particles (with minimum 100 Å and maximum 200 Å) were selected after two rounds of 2D classification, re-extracted for recentering, and used to generate ab initio 3D volumes. These volumes were then subjected to heterogeneous refinement with three classes. Particles belonging to the best-looking class were re-extracted for centering and used for ab initio constructions with two classes. Particles from the best-looking volume underwent non-uniform refinement and were then subjected to 2D classification. The newly generated 2D projections were used for template-based particle picking (particle diameter: 100 Å), and the extracted particles were filtered through two rounds of 2D classification. Selected particles were used to train a Topaz model^11^ (particle diameter: 100 Å, particle number: 300). Topaz extract was performed (particle diameter: 100 Å), and particles were further selected through two rounds of 2D classification. Particles from three separate particle detection methods were combined, with duplicate particles removed with a minimum distance of 100 Å. The remaining unique particles were used for ab initio reconstruction with six classes, and the best-looking class, marked with a dashed box, was further processed with non-uniform refinement. This resulted in the best resolution of 6.7 Å, as shown in the FSC curves, with a slight orientational preference of the side views. This map has been deposited to EMDB with the code EMD-48154. 3D classification without alignment isolated three classes marked with dashed boxes showing auxiliary density. The final volume after non-uniform refinement revealed a more pronounced density of the auxiliary particle, although the final resolution was slightly lower at 6.93 Å.

TMD and CTD subunit cores obtained from Alphafold Protein Structure Database^12^ were manually fit to the density map contoured at the high density of 0.34 for each subunit individually (Fig. 5B). Human dynein light chain dimer (3E2B)^13^ complexed with two binding motifs to each monomer was manually fit to the additional density (Fig. 5C).

**References**

1. Wiśniewski, J.R., Zougman, A., Nagaraj, N. & Mann, M. Universal sample preparation method for proteome analysis. *Nat Methods* **6**, 359-62 (2009).

2. Rappsilber, J., Mann, M. & Ishihama, Y. Protocol for micro-purification, enrichment, pre-fractionation and storage of peptides for proteomics using StageTips. *Nat Protoc* **2**, 1896-906 (2007).

3. Contrepois, K., Ezan, E., Mann, C. & Fenaille, F. Ultra-high performance liquid chromatography-mass spectrometry for the fast profiling of histone post-translational modifications. *J Proteome Res* **9**, 5501-9 (2010).

4. Perkins, D.N., Pappin, D.J., Creasy, D.M. & Cottrell, J.S. Probability-based protein identification by searching sequence databases using mass spectrometry data. *Electrophoresis* **20**, 3551-67 (1999).

5. Perez-Riverol, Y. et al. The PRIDE database at 20 years: 2025 update. *Nucleic Acids Res* **53**, D543-D553 (2025).

6. Teyra, J. et al. Large-scale survey and database of high affinity ligands for peptide recognition modules. *Mol Syst Biol* **16**, e9310 (2020).

7. Kang, C. et al. SWELL1 is a glucose sensor regulating β-cell excitability and systemic glycaemia. *Nat Commun* **9**, 367 (2018).

8. Gunasekar, S.K. et al. Small molecule SWELL1 complex induction improves glycemic control and nonalcoholic fatty liver disease in murine Type 2 diabetes. *Nat Commun* **13**, 784 (2022).

9. Lee, S.J., Maeda, S., Gao, J. & Nichols, C.G. Oxidation Driven Reversal of PIP_2_-dependent Gating in GIRK2 Channels. *Function (Oxf)* **4**, zqad016 (2023).

10. Punjani, A., Rubinstein, J.L., Fleet, D.J. & Brubaker, M.A. cryoSPARC: algorithms for rapid unsupervised cryo-EM structure determination. *Nat Methods* **14**, 290-296 (2017).

11. Bepler, T., Kelley, K., Noble, A.J. & Berger, B. Topaz-Denoise: general deep denoising models for cryoEM and cryoET. *Nat Commun* **11**, 5208 (2020).

12. Tunyasuvunakool, K. et al. Highly accurate protein structure prediction for the human proteome. *Nature* **596**, 590-596 (2021).

13. Benison, G., Karplus, P.A. & Barbar, E. The interplay of ligand binding and quaternary structure in the diverse interactions of dynein light chain LC8. *J Mol Biol* **384**, 954-66 (2008).
