# Supplementary material for: Dynein light chains 1 and 2 are auxiliary proteins of pH-sensitive Kir4.1 channels
Source: J Biol Chem. 2025 Mar 10;301(4):108393. doi: 10.1016/j.jbc.2025.108393 (PMC11999606; doi:10.1016/j.jbc.2025.108393)
Supplement: SupportingInformation_Table [file mmc4.docx]

**Dynein light chains 1 and 2 are auxiliary proteins of pH-sensitive Kir4.1 channels**

# Sun-Joo Lee^1,#^, Jian Gao^1^, Ellen Thompson^1^, Jonathan Mount^2^, and Colin G. Nichols^1^

^1^Department of Cell Biology and Physiology and the Center for Investigation of Membrane Excitability Diseases, Washington University School of Medicine, St. Louis, Missouri, USA,

^2^Department of Anesthesiology, Weill Cornell Medical College, New York, NY, USA

^#^ To whom correspondence should be addressed.

**Supporting Information Table ST1.**

The full list of proteins observed in the four samples shown in Supp. Inf. SF. 1 and Fig. 1D. The peptides were filtered with 95% peptide thresholds and 0.1% Protein FDR. The proteins were ordered by their % occupancy in the Whole sample.
